# Supplementary material for: Identification of slit3 as a locus affecting nicotine preference in zebrafish and human smoking behaviour
Source: eLife. 2020 Mar 25;9:e51295. doi: 10.7554/eLife.51295 (PMC7096180; doi:10.7554/eLife.51295)
Supplement: Supplementary file 1. — Supplementary methods: (A) Loss of function mutations present in AJBQM1 and AJBQM2 founders; (B) Gene identifiers and primer sequences used for zebrafish gene expression analysis; (C) Human association study sample characteristics; (D) Primer and reporter sequences used for human genotyping. Supplementary results: (H) Results of site specific PCR genotyping of AJBQM1 and AJBQM2; (F) Logistic regression analysis for human association analysis on smoking initiation; (G) Logistic regression analysis for human association analysis on persistent smoking; (H) Full qPCR gene expression results for slit3 wildtype and slit3sa1569/sa1569 homozygous mutants. [file elife-51295-supp1.docx]

**Supplementary Table 1:**

**A)**

**
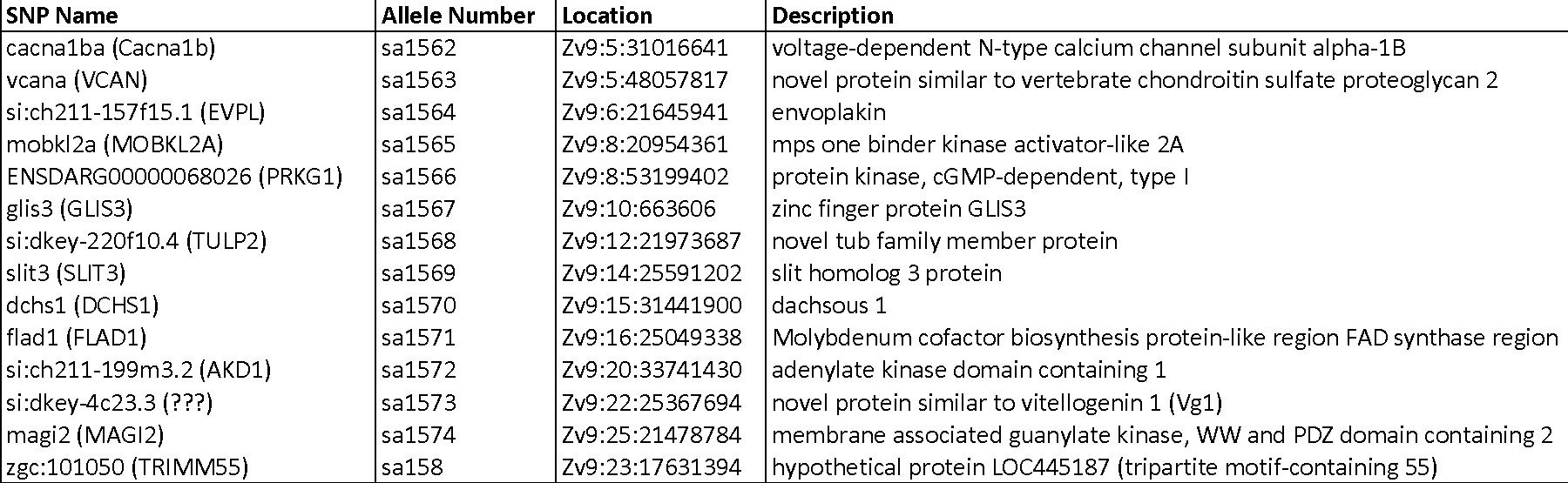
**

**B)**


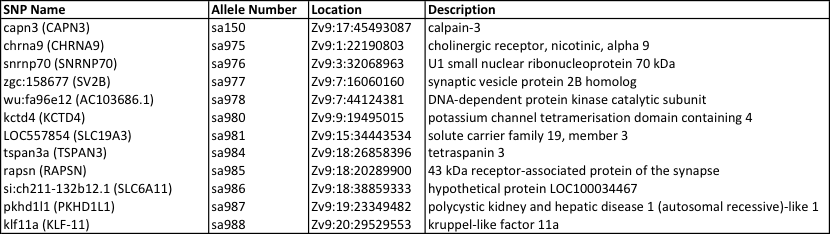


**Supplementary Table 2**.

| Gene name | Transcript ID | Sequence (5’ -> 3’) | Fragment size (bp) |
| --- | --- | --- | --- |
| *Serotonergic pathway* | | | |
| *htr1aa* | NM_001123321.1 | Forward: TTCTACATCCCGCTCATCCTCA | 180 |
|  |  | Reverse: CCTCCAAGTTTTACCCACCTCTC |  |
| *htr1ab* | NM_001145766.1 | Forward: AAACACCGAGGCGAAGAGGAA | 99 |
|  |  | Reverse: GGCAGCCAACACAGAATGAAAGT |  |
| *htr2a* | XM_684208.9 | Forward: TACGGTGGCTGGGAACATTTTAG | 187 |
|  |  | Reverse: GGGACACAGTGATGCAGGGAAA |  |
| *htr5a* | NM_001126410.2 | Forward: TGGATCAAAGAGGACCAACACC | 118 |
|  |  | Reverse: CTGAAACGTCACCGTGGCAT |  |
| *htr2cl1* | NM_001129893.1 | Forward: AACTTCTTCCTCCGCTCACTCG | 179 |
|  |  | Reverse: ATGGCACACAGGTGCATGATGG |  |
| *htr2cl2* | XM_001339004.7 | Forward: CACAACCCCACCAACTTCTTCC | 152 |
|  |  | Reverse: ACGTCCAGAAAGATCCACAGCG |  |
| *htr4* | XM_021481160.1 XM_009291062.3 | Forward: GTTTCTTTCCAAGCGCCTC | 168 |
|  |  | Reverse: ACTTCTTCCATCTCAGGCATC |  |
| *htr6* | XM_009297078.3 | Forward: ACTACAGTCATCAGGAGCCACC | 147 |
|  |  | Reverse: GCCAGGCACTGAAGAATAGTCC |  |
| *htr7* | XM_003199584.5 | Forward: TGGATGTGATGTGCTGTACCGC | 118 |
|  |  | Reverse: GCCATGCACTTTCCACTCTGTC |  |
| *slc6a4/ SERT* | NM_001039972.1 | Forward: ACCAGGGGCGAAGCCAAGCA | 117 |
|  |  | Reverse: GCCACAGGCCCCGCTGTTA |  |
| *htr1b* | NM_001128709.1 | Forward: CCTTGTCGTCAGTTCTGGGT | 112 |
|  |  | Reverse: ATCAGAAAGTTCGCCGGTGT |  |
| *Nicotinic pathway* | | |  |
| *chrna2* | NC_007128.7 | Forward: TGGCTGCAGATCAGTCAAAGAC | 271 |
|  |  | Reverse: CCCTCTAACTGTCCCTTCACAA |  |
| *chrna3* | Not found in BLAST | Forward: TGTACATCCGCCGATTACCGCT | ? |
|  |  | Reverse: TCCGCAGTCGGAGGGCAGTA |  |
| *chrna4b* | ENSDART00000018614.7 | Forward: TTACAAGAGGTTTGGGCGCT | 90 |
|  |  | Reverse: ACAGACCAGTAGATCATCACTCC |  |
| *chrna5* | NM_001017885.1 | Forward: GGCTCCCAGGTCGACATTCTC | 103 |
|  |  | Reverse: AACCCCGGTTACCAGTGGCCT |  |
| *chrna6* | NM_001042684 | Forward: AGGCTCTTTCGTCGTTTATTC | 156 |
|  |  | Reverse: TCTCAGCCAAAGGTTTGTTTC |  |
| *chrna7* | ENSDART00000171463.3 and ENSDART00000166391.2 | Forward: ACCGTGTCACATTGTTCATTCTC | 105 |
|  |  | Reverse: ACAGGTCTCTCCAGTGGGTTA |  |
| *chrnb2b* | ENSDART00000041625.7 and ENSDART00000185728.1 | Forward: CACAAAGTCACGCTCCGATAC | 160 |
|  |  | Reverse: CCGTCGCTCTGAGCAGATAA |  |
| *chrnb3b* | ENSDARG00000038508.5 | Forward: CAGGAGTCAACCTCCGCTTT | 106 |
|  |  | Reverse: TGAATCTGAACGCACTGGCT |  |
| *chrnb4* | NC_007129.7 | Forward: ATGTGAATGAATGGCGGTGTGTG | 203 |
|  |  | Reverse: ATGCGCGTGTCAGATTTACCC |  |
| *Dopaminergic pathway* | | |  |
| *drd1b* | NM_001135976.2 | Forward: TGGTTCCTTTCTGCAACCCA | 100 |
|  |  | Reverse: AGTGATGAGTTCGCCCAACC |  |
| *drd2a* | XM_009291617.3 and XM_005157501.4 | Forward: TCCACAAAATCAGGAAAAGCGT | 106 |
|  |  | Reverse: CAGCCAATGTAAACCGGCAA |  |
| *drd3* | XM_021470111.1 and XM_005162673.4 | Forward: ATCGAGTTTCGCAGAGCCTT | 95 |
|  |  | Reverse: TCCACAGTGTCTGAAAGCCG |  |
| *slc6a3* | NM_131755.1 | Forward: GCCTGGTTTTACGGAGTGGA | 66 |
|  |  | Reverse: GGAGGATTGAAGGTGGCGAA |  |
| *Adrenergic pathway* | | |  |
| *adra1aa* | NM_001324454.1 | Forward: AAGAAGGCCGCCAAGACTTT | 114 |
|  |  | Reverse: GTCCGAGGGTCTGTACGTTG |  |
| *adra1d* | XM_691951.6 | Forward: AAGCTGCTAAAACCCTCGCC | 103 |
|  |  | Reverse: GGCTTCAGAGCTGGGAAGAAT |  |
| *adra2b* | NM_207638.1 | Forward: AAAAGCCAGGCCTCCAACTT | 92 |
|  |  | Reverse: GGGCTTGCAGAAGGTTGTTG |  |
| *adra2*c | NM_207639.1 | Forward: CGCCGTTTTAACGAGCAGAG | 87 |
|  |  | Reverse: AGTGTGGCCACCAGAATGTC |  |
| *adra2da* | NM_194364.2 | Forward: CATCATCCTCGTGGTGTCCC | 188 |
|  |  | Reverse: ATCCCATGATCTCGTTGGCG |  |
| *adra2db* | NM_194365.1 | Forward: TGCCACTTTGGTCATTCCGT | 88 |
|  |  | Reverse: AGCCAGGTAGAAAGCACACC |  |

**Supplementary Table 3:**

| ***Cohort name*** | ***N*** | ***Country*** | ***Cohort description*** | ***ClinicalTrials.gov ID*** | ***Mean age (years)*** | ***% female*** | ***Smoking phenotypes investigated*** | ***N heavy smokers*** |
| --- | --- | --- | --- | --- | --- | --- | --- | --- |
| ViDiCO | 272 | UK | Subjects with mild, moderate or severe chronic obstructive pulmonary disease (COPD) treated with the same bi-monthly 3mg vitamin D3 intervention. | NCT00977873 | 64.6 | 40 | Tobacco consumption; smoking cessation (current vs ever smokers) | 249 |
| ViDiAs | 293 | UK | Adult patients with asthma treated with inhaled corticosteroids treated with a bi-monthly 3mg vitamin D3 intervention | NCT00978315 | 47 | 56 | Tobacco consumption; smoking cessation (current vs ever smokers) | 17 |
| ViDiFLU | 298 | UK | Adults in sheltered accommodation given 10 mcg vitamin D3 daily as well as bi-monthly 3mg vitamin D3 interventions | NCT01069874 | 66.8 | 66 | Tobacco consumption; smoking cessation (current vs ever smokers) | 66 |
| Finnish Twins | 1915, 1715, 1726* | Finland | Study sample ascertained from the Finnish Twin Cohort study (N=35834 adult twins) concordant for moderate to heavy smoking | NA | 55 | 48 | DSM-IV nicotine dependence symptoms; Fagerström scores; cigarettes smoked each day; sensation felt after smoking first cigarette and time to first cigarette in the morning | NA |

**Supplementary Table 4**.

| **GENE** | **SNP** | **Sequence name** | **Sequence** |
| --- | --- | --- | --- |
| *CYP3A4* | rs2740574 |  | |
|  |  | Forward | CCAGGCATAGGTAAAGATCTGTAGGT |
|  |  | Reverse | CTCAAGTGGAGCCATTGGCATA |
|  |  | Reporters | ACAAGGGCAAGAGAG and ACAAGGGCAGGAGAG |
| *CUBN* | rs3740165 |  | |
|  |  | Forward | GCAATGAGATTAAATCTTCAGGAAACACA |
|  |  | Reverse | CTGGAGGTATAGGAAGCAGTGAAG |
|  |  | Reporters | CCGCCATATGGCCTG and CGCCATACGGCCTG |
| *RXRA* | rs7861779 |  | |
|  |  | Forward | TGGCCCATGCACGAGTAG |
|  |  | Reverse | ACCGAGACAGGCCAAACTC |
|  |  | Reporters | CAGCAGAGGTGGCCGA and CAGCAGAGATGGCCGA |

**Supplementary Table 5:**

**(A)**

|  |  | Gene name | | | | | | | | | | | | |
| --- | --- | --- | --- | --- | --- | --- | --- | --- | --- | --- | --- | --- | --- | --- |
| **Fish** | **CPP Change Score** | ***slit3*** | *cacne* | *vcan* | *evpl* | *mob3a* | *prkg1* | *glis3* | *tulp2* | *dchs1* | *flad1* | *akd1* | *magI2* | *trimm55* |
| **1** | **0** | **WT** | WT | HET | WT | HET | HET | WT | HOM | WT | HOM | HOM | HET | WT |
| **2** | **0.01** | **WT** | HET | HET | WT | HET | HET | HET | HOM | WT | HOM | HOM | WT | HET |
| **3** | **0.07** | **WT** | WT | WT | WT | HET | HET | HET | WT | WT | WT | WT | HET | WT |
| **4** | **0.15** | **WT** | WT | HET | WT | HOM | HET | HET | HET | WT | HET | HET | WT | HET |
| **5** | **0.32** | **HET** | HOM | HOM | WT | HOM | HET | WT | WT | WT | WT | WT | HET | WT |
| **6** | **0.43** | **HET** | HET | WT | WT | HET | HET | WT | HET | WT | WT | HET | HOM | WT |
| **7** | **0.44** | **HET** | WT | HET | WT | WT | HET | WT | HET | WT | WT | HET | WT | WT |
| **8** | **0.47** | **HET** | WT | HET | WT | HET | HET | HET | HET | WT | HET | HET | WT | WT |
| **9** | **0.51** | **HET** | WT | WT | WT | WT | HOM | WT | WT | WT | HET | WT | HOM | HET |
| **10** | **0.6** | **HET** | WT | WT | WT | HET | HOM | HET | WT | WT | HET | WT | HET | WT |
| ***P-value*** | | **7.66x10^-5^** | 0.691 | 0.259 | - | 0.236 | - | 0.602 | 0.481 | - | 0.981 | 0.418 | 0.73 | 0.51 |

**(B)**

|  |  | Gene name | | | | | | | | | | | |
| --- | --- | --- | --- | --- | --- | --- | --- | --- | --- | --- | --- | --- | --- |
| **Fish number** | **CPP Change Scores** | *tspan3a* | *raspn* | *a9* | *capn3* | *klf11a* | *kctd4* | *slc6a11* | *pkhd1l1* | *slc19a3* | *sv2b* | *snrnp70* | *ac10103686* |
| **1** | **-0.38** | HET | WT | WT | HET | WT | HET | HET | WT | WT | WT | WT | WT |
| **2** | **-0.28** | HET | HOM | WT | HET | WT | WT | HET | WT | WT | WT | WT | WT |
| **3** | **-0.27** | HET | WT | HET | HET | WT | WT | HET | WT | WT | HET | WT | WT |
| **4** | **-0.23** | WT | WT | WT | WT | HET | HET | WT | HET | WT | WT | WT | WT |
| **5** | **-0.21** | WT | WT | WT | HET | WT | WT | HET | HOM | WT | WT | WT | WT |
| **6** | **-0.18** | WT | HOM | WT | WT | WT | WT | WT | HOM | WT | WT | WT | WT |
| **7** | **-0.17** | HET | HOM | HET | WT | WT | HET | HET | HET | WT | WT | WT | WT |
| **8** | **-0.17** | HET | HOM | WT | HET | HET | WT | HET | WT | WT | HET | WT | WT |
| **9** | **-0.12** | WT | WT | HET | WT | WT | HET | WT | WT | WT | HOM | WT | WT |
| **10** | **-0.09** | WT | WT | HET | HET | WT | HET | WT | WT | WT | HET | WT | WT |
| **11** | **-0.09** | WT | WT | HET | WT | HET | HET | WT | WT | WT | HOM | WT | WT |
| **12** | **-0.07** | HET | HOM | HET | WT | WT | HET | WT | WT | WT | HOM | WT | WT |
| **13** | **0.04** | HET | WT | HET | WT | HET | WT | HET | WT | WT | HET | WT | WT |
| **14** | **0.07** | HET | HOM | HET | WT | WT | WT | WT | HOM | WT | HOM | WT | WT |
|  | **P-value** | 0.583 | 0.792 | 0.339 | 0.911 | 0.318 | 0.252 | 0.697 | 0.499 | - | 0.269 | - | - |

**Supplementary Table 6:**

**
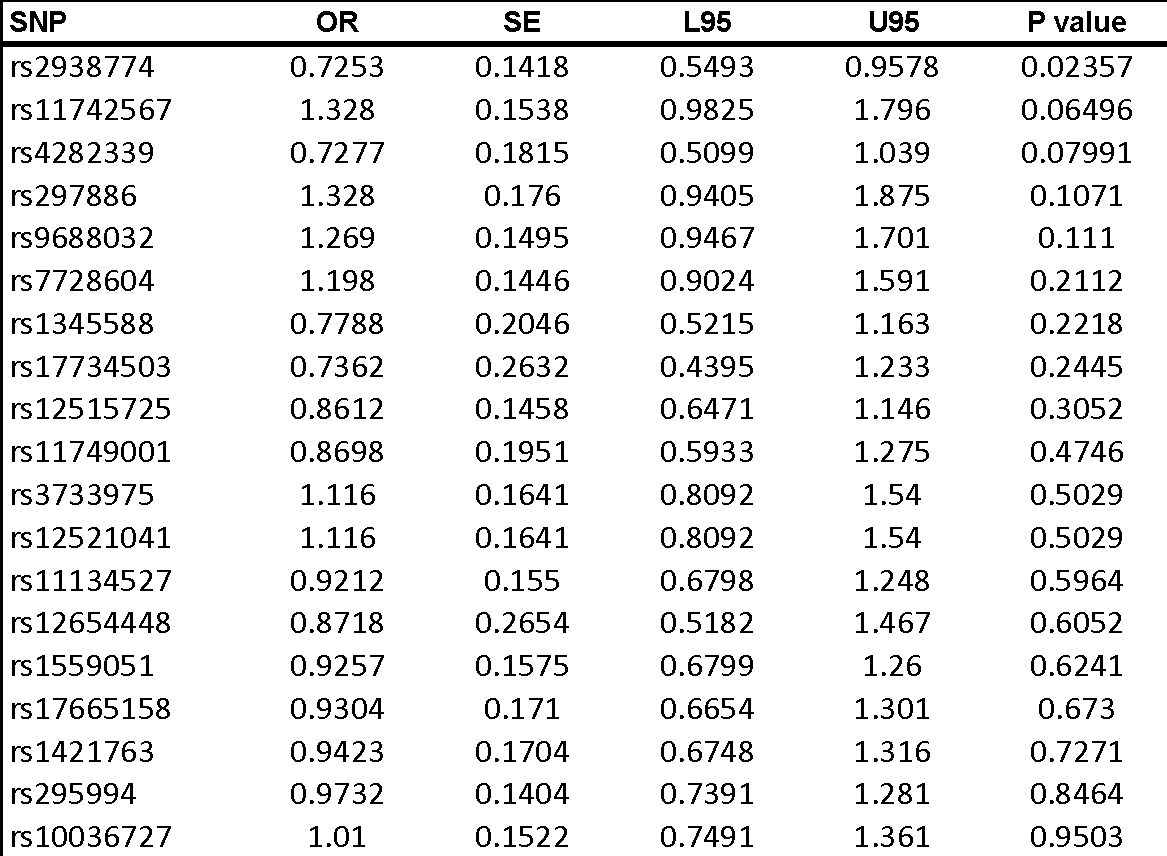
**

**Supplementary Table 7:**

**
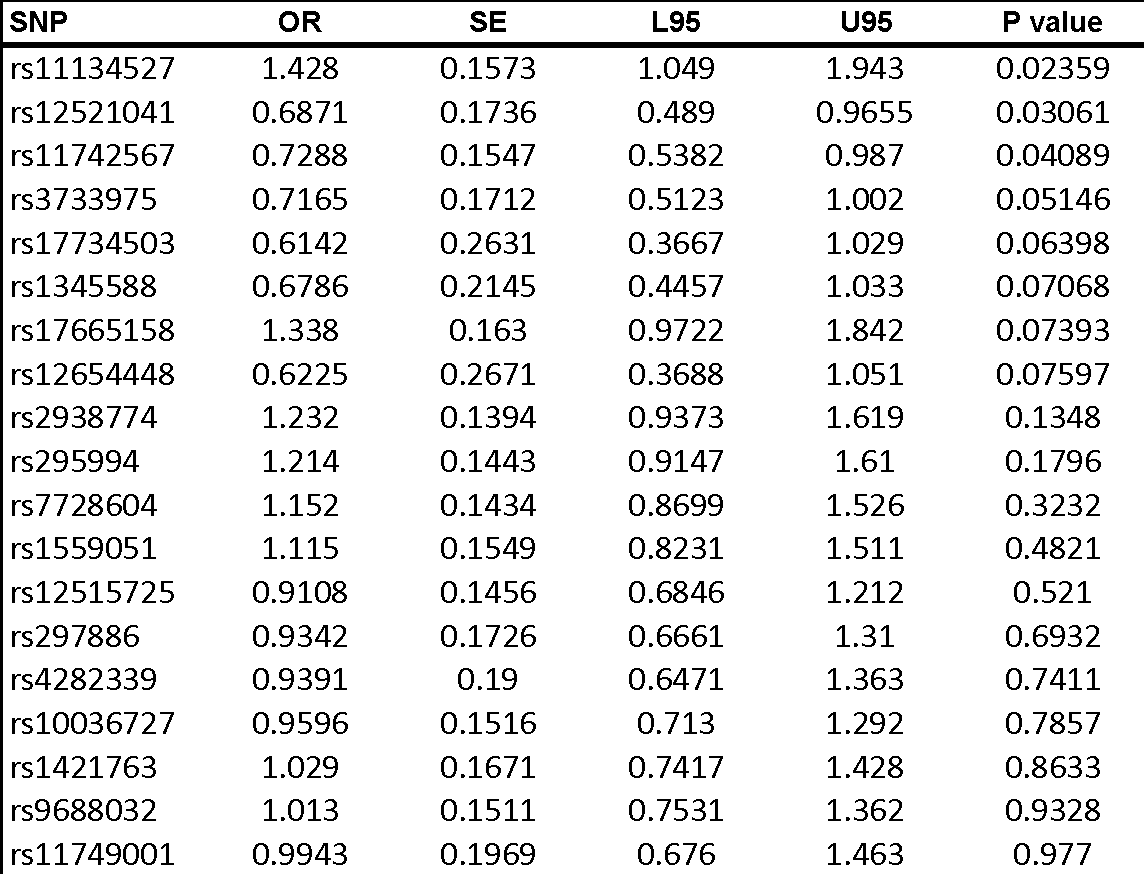
**

**Supplementary Table 8**.

| **Gene name** | ***Mean (SD)***  ***Slit3^+/+^*** | ***Mean (SD)***  ***Slit3^sa1569/-^*** | ***Mean (SD)***  ***Slit3^sa1569/sa1569^*** | **F (DFn, DFd)** | ***p*** |
| --- | --- | --- | --- | --- | --- |
| *Serotonergic pathway* | | | | | |
| ***htr1aa*** | **5.30 x 10^-4^** | **1.43 x 10^-3^** | **1.27 x 10^-3^** | **F (2, 6) = 44** | **0.0003** |
|  | **(2.60 x 10^-5^)** | **(1.53 x 10^-4^)** | **(1.53 x 10^-4^)** |  |  |
| *htr1ab* | 8.63 x 10^-4^ | 9.30 x 10^-4^ | 9.23 x 10^-4^ | F (2, 6) = 0.2594 | 0.7797 |
|  | (1.25 x 10^-4^) | (1.54 x 10^-4^) | (8.62 x 10^-5^) |  |  |
| *htr2a* | 5.87 x 10^-4^ | 7.43 x 10^-4^ | 5.73 x 10^-4^ | F (2, 6) = 0.8987 | 0.4556 |
|  | (1.05 x 10^-4^) | (2.35 x 10^-4^) | (1.53 x 10^-4^) |  |  |
| *htr5a* | 2.90 x 10^-5^ | 5.20 x 10^-5^ | 4.73 x 10^-5^ | F (2, 6) = 0.4694 | 0.6465 |
|  | (2.20 x 10^-5^) | (4.22 x 10^-5^) | (2.41 x 10^-5^) |  |  |
| *htr2cl1* | 1.13 x 10^-3^ | 1.43 x 10^-3^ | 1.33 x 10^-3^ | F (2, 6) = 1.500 | 0.2963 |
|  | (1.16 x 10^-4^) | (3.22 x 10^-4^) | (1.53 x 10^-4^) |  |  |
| *htr2cl2* | 3.87 x 10^-5^ | 6.50 x 10^-5^ | 5.17 x 10^-5^ | F (2, 6) = 0.9413 | 0.4410 |
|  | (5.13 x 10^-6^) | (3.21 x 10^-5^) | (2.46 x 10^-5^) |  |  |
| *htr4* | 1.02 x 10^-3^ | 1.40 x 10^-3^ | 1.43 x 10^-3^ | F (2, 6) = 7.654 | 0.0223 |
|  | (1.33 x 10^-4^) | (2.00 x 10^-4^) | (5.77 x 10^-5^) |  |  |
| *htr6* | 1.50 x 10^-5^ | 6.17 x 10^-5^ | 4.23 x 10^-5^ | F (2, 6) = 4.184 | 0.0728 |
|  | (2.00 x 10^-6^) | (3.26 x 10^-5^) | (1.07 x 10^-5^) |  |  |
| *htr7* | 1.23 x 10^-4^ | 1.12 x 10^-4^ | 1.73 x 10^-4^ | F (2, 6) = 0.8930 | 0.4576 |
|  | (3.11 x 10^-5^) | (5.35 x 10^-5^) | (8.39 x 10^-5^) |  |  |
| *slc6a4/ sert* | 1.40 x 10^-3^ | 2.00 x 10^-3^ | 1.83 x 10^-3^ | F (2, 6) = 3.408 | 0.1026 |
|  | (1.00 x 10^-4^) | (4.36 x 10^-4^) | (2.31 x 10^-4^) |  |  |
| *htr1b* | 9.53 x 10^-4^ | 1.33 x 10^-3^ | 1.13 x 10^-3^ | F (2, 6) = 1.617 | 0.2744 |
|  | (4.51 x 10^-5^) | (4.16 x 10^-4^) | (1.62 x 10^-4^) |  |  |
| *Overall serotonergic* | *6.09 x 10^-4^* | *8.69 x 10^-4^* | *8.00 x 10^-4^* | *F (2, 30) = 0.5137* | *0.6034* |
|  | *(5.01 x 10^-4^)* | *(7.05 x 10^-4^)* | *(6.51 x 10^-4^)* |  |  |
| *nicotinic pathway* | | | | | |
| *chrna2* | 1.49 x 10^-5^ | 1.60 x 10^-5^ | 3.80 x 10^-7^ | F (2, 6) = 0.8431 | 0.4757 |
|  | (6.47 x 10^-6^) | (2.77 x 10^-5^) | (2.82 x 10^-7^) |  |  |
| *chrna3* | 3.53 x 10^-4^ | 4.13 x 10^-4^ | 3.70 x 10^-4^ | F (2, 6) = 1.044 | 0.4081 |
|  | (5.03 x 10^-5^) | (4.62 x 10^-5^) | (6.00 x 10^-5^) |  |  |
| *chrna4b* | 4.13 x 10^-4^ | 6.10 x 10^-4^ | 5.73 x 10^-4^ | F (2, 6) = 5.836 | 0.0391 |
|  | (4.51 x 10^-5^) | (1.05 x 10^-4^) | (6.11 x 10^-5^) |  |  |
| *chrna5* | 1.43 x 10^-3^ | 2.00 x 10^-3^ | 1.90 x 10^-3^ | F (2, 6) = 2.906 | 0.1311 |
|  | (1.53 x 10^-4^) | (3.61 x 10^-4^) | (3.61 x 10^-4^) |  |  |
| *chrna6* | 2.13 x 10^-3^ | 2.63 x 10^-3^ | 2.23 x 10^-3^ | F (2, 6) = 2.333 | 0.178 |
|  | (5.77 x 10^-5^) | (5.03 x 10^-4^) | (1.16 x 10^-4^) |  |  |
| *chrna7* | 5.75 x 10^-5^ | 8.53 x 10^-5^ | 7.50 x 10^-5^ | F (2, 6) = 0.4101 | 0.6808 |
|  | (5.04 x 10^-5^) | (4.15 x 10^-5^) | (8.72 x 10^-6^) |  |  |
| *chrnb2b* | 5.20 x 10^-4^ | 9.67 x 10^-4^ | 8.40 x 10^-4^ | F (2, 6) = 18.18 | 0.0028 |
|  | (2.00 x 10^-5^) | (1.53 x 10^-4^) | (5.00 x 10^-5^) |  |  |
| *chrnb3b* | 6.27 x 10^-6^ | 8.73 x 10^-5^ | 1.45 x 10^-4^ | F (2, 6) = 5.870 | 0.0387 |
|  | (3.51 x 10^-6^) | (2.47 x 10^-5^) | (8.26 x 10^-5^) |  |  |
| *chrnb4* | 4.70 x 10^-5^ | 7.57 x 10^-5^ | 5.93 x 10^-5^ | F (2, 6) = 0.8718 | 0.4652 |
|  | (9.17 x 10^-6^) | (3.84 x 10^-5^) | (2.40 x 10^-5^) |  |  |
| *Overall nicotinic* | *5.53 x 10^-4^* | *7.65 x 10^-4^* | *6.89 x 10^-4^* | *F (2, 24) = 0.1455* | *0.8654* |
|  | *(7.44 x 10^-4^)* | *(9.46 x 10^-4^)* | *(8.32 x 10^-4^)* |  |  |
| *dopaminergic pathway* | | | | | |
| *drd1b* | 1.65 x 10^-3^ | 2.15 x 10^-3^ | 2.01 x 10^-3^ | F (2, 6) = 4.333 | 0.0685 |
|  | (2.46 x 10^-4^) | (2.66 x 10^-4^) | (7.00 x 10^-5^) |  |  |
| *drd2a* | 6.42 x 10^-4^ | 8.72 x 10^-4^ | 8.29 x 10^-4^ | F (2, 6) = 1.484 | 0.2995 |
|  | (1.25 x 10^-4^) | (2.51 x 10^-4^) | (1.11 x 10^-4^) |  |  |
| *drd3* | 1.30 x 10^-3^ | 1.53 x 10^-3^ | 1.60 x 10^-3^ | F (2, 6) = 5.154 | 0.0498 |
|  | (1.00 x 10^-4^) | (1.53 x 10^-4^) | (1.00 x 10^-4^) |  |  |
| *slc6a3* | 5.43 x 10^-4^ | 5.80 x 10^-4^ | 6.60 x 10^-4^ | F (2, 6) = 4.089 | 0.0758 |
|  | (3.51 x 10^-5^) | (6.25 x 10^-5^) | (5.20 x 10^-5^) |  |  |
| *Overall dopaminergic* | *1.04 x 10^-3^* | *1.28 x 10^-3^* | *1.28 x 10^-3^* | *F (2, 9) = 0.2025* | *0.8203* |
|  | *(5.32 x 10^-4^)* | *(7.02 x 10^-4^)* | *(6.39 x 10^-4^)* |  |  |
| *adrenergic pathway* | | | | | |
| *adra1aa* | 1.20 x 10^-3^ | 1.70 x 10^-3^ | 1.57 x 10^-3^ | F (2, 6) = 4.892 | 0.0549 |
|  | (1.73 x 10^-4^) | (2.65 x 10^-4^) | (1.53 x 10^-4^) |  |  |
| *adra1d* | 8.30 x 10^-4^ | 9.43 x 10^-4^ | 8.63 x 10^-4^ | F (2, 6) = 0.2954 | 0.7545 |
|  | (8.72 x 10^-5^) | (2.89 x 10^-4^) | (1.10 x 10^-4^) |  |  |
| *adra2b* | 3.93 x 10^-3^ | 5.23 x 10^-3^ | 4.43 x 10^-3^ | F (2, 6) = 12.90 | 0.0067 |
|  | (2.52 x 10^-4^) | (3.79 x 10^-4^) | (3.06 x 10^-4^) |  |  |
| *adra2c* | 1.30 x 10^-3^ | 1.60 x 10^-3^ | 1.50 x 10^-3^ | F (2, 6) = 3.500 | 0.0983 |
|  | (1.00 x 10^-4^) | (2.00 x 10^-4^) | (1.00 x 10^-4^) |  |  |
| *adra2da* | 3.27 x 10^-4^ | 4.17 x 10^-4^ | 4.00 x 10^-4^ | F (2, 6) = 3.005 | 0.1247 |
|  | (2.31 x 10^-5^) | (7.51 x 10^-5^) | (2.65 x 10^-5^) |  |  |
| *adra2db* | 2.90 x 10^-4^ | 2.87 x 10^-4^ | 3.57 x 10^-4^ | F (2, 6) = 0.6630 | 0.5494 |
|  | (9.54 x 10^-5^) | (8.02 x 10^-5^) | (7.51 x 10^-5^) |  |  |
| *Overall adrenergic* | *1.31 x 10^-3^* | *1.70 x 10^-3^* | *1.52 x 10^-3^* | *F (2, 15) = 0.08865* | *0.9156* |
|  | *(1.35 x 10^-3^)* | *(1.83 x 10^-3^)* | *(1.52 x 10^-3^)* |  |  |
