# Supplementary material for: Identification of slit3 as a locus affecting nicotine preference in zebrafish and human smoking behaviour
Source: eLife. 2020 Mar 25;9:e51295. doi: 10.7554/eLife.51295 (PMC7096180; doi:10.7554/eLife.51295)
Supplement: Transparent reporting form [file elife-51295-transrepform.docx.pdf]

1st Floor
24 Hills Road
Cambridge CB2 1JP, UK

P 01223 855340
W elifesciences.org
T @elife

eLife's transparent reporting form

We encourage authors to provide detailed information *within their submission* to facilitate the interpretation and replication of experiments. Authors can upload supporting documentation to indicate the use of appropriate reporting guidelines for health-related research (see [EQUATOR Network]), life science research (see the [BioSharing Information Resource]), or the [ARRIVE guidelines] for reporting work involving animal research. Where applicable, authors should refer to any relevant reporting standards documents in this form.

If you have any questions, please consult our Journal Policies and/or contact us: editorial@elifesciences.org.

Sample-size estimation

- You should state whether an appropriate sample size was computed when the study was being designed
- You should state the statistical method of sample size computation and any required assumptions
- If no explicit power analysis was used, you should describe how you decided what sample (replicate) size (number) to use

Please outline where this information can be found within the submission (e.g., sections or figure legends), or explain why this information doesn't apply to your submission:

- For animals: Methods. Section called "Animals"
- For humans: Supplementary methods. Supplementary Table 3

Replicates

- You should report how often each experiment was performed
- You should include a definition of biological versus technical replication
- The data obtained should be provided and sufficient information should be provided to indicate the number of independent biological and/or technical replicates
- If you encountered any outliers, you should describe how these were handled
- Criteria for exclusion/inclusion of data should be clearly stated
- High-throughput sequence data should be uploaded before submission, with a private link for reviewers provided (these are available from both GEO and ArrayExpress)

Please outline where this information can be found within the submission (e.g., sections or figure legends), or explain why this information doesn't apply to your submission:

- ***For CPP forward genetics screen***: Methods. Section called Conditioned place preference (CPP). Exact sample sizes for each experimental group specified in Figure legends 1-4
- ***For antibody staining and confocal imaging****:* Figure legend 5.
- ***For startle response****:* Methods. Section called startle response in the presence or absence of amisulpride.
- ***For qPCR***. Methods. Section called Real-time quantitative PCR.
- ***For human analyses***. Methods. Section called Human cohorts.

eLife Sciences Publications, Ltd is a limited liability non-profit non-stock corporation incorporated in the State of Delaware, USA, with company number 5030732, and is registered in the UK with company number FC030576 and branch number BR015634 at the address 1st Floor, 24 Hills Road, Cambridge CB2 1JP | August 2014

Statistical reporting

- Statistical analysis methods should be described and justified
- Raw data should be presented in figures whenever informative to do so (typically when N per group is less than 10)
- For each experiment, you should identify the statistical tests used, exact values of N, definitions of center, methods of multiple test correction, and dispersion and precision measures (e.g., mean, median, SD, SEM, confidence intervals; and, for the major substantive results, a measure of effect size (e.g., Pearson's r, Cohen's d)
- Report exact p-values wherever possible alongside the summary statistics and 95% confidence intervals. These should be reported for all key questions and not only when the p-value is less than 0.05.

Please outline where this information can be found within the submission (e.g., sections or figure legends), or explain why this information doesn't apply to your submission:

- ***For Conditioned Place Preference:*** Statistical analysis in Methods. Subsection called 'Data analysis' within Conditioned place preference.
- ***For antibody staining and confocal imaging****:* No statistical assessment was performed
- ***For Startle response****. Figure legend 6*
- ***For qPCR****. Figure legend 8*
- ***For human analyses****. Table legend 1 and table legend 2*

(For large datasets, or papers with a very large number of statistical tests, you may upload a single table file with tests, Ns, etc., with reference to sections in the manuscript.)

Group allocation

- Indicate how samples were allocated into experimental groups (in the case of clinical studies, please specify allocation to treatment method); if randomization was used, please also state if restricted randomization was applied
- Indicate if masking was used during group allocation, data collection and/or data analysis

Please outline where this information can be found within the submission (e.g., sections or figure legends), or explain why this information doesn't apply to your submission:

- ***For Conditioned Place Preference:*** Methods. Section called Conditioned place preference (CPP) specifies how fish were allocated to be conditioned to spots or stripes.
- ***For antibody staining and confocal imaging***. Experimental group allocated by genotype. Specified in Methods
- ***For Startle response*** Experimental group allocated by genotype. Specified in Methods
- ***For qPCR*** Experimental group allocated by genotype. Specified in Methods
- ***For human analyses*** Allocation of experimental groups in supplementary material and Supplementary Table 3.

Additional data files ("source data")

- We encourage you to upload relevant additional data files, such as numerical data that are represented as a graph in a figure, or as a summary table
- Where provided, these should be in the most useful format, and they can be uploaded as "Source data" files linked to a main figure or table
- Include model definition files including the full list of parameters used
- Include code used for data analysis (e.g., R, MatLab)

eLife Sciences Publications, Ltd is a limited liability non-profit non-stock corporation incorporated in the State of Delaware, USA, with company number 5030732, and is registered in the UK with company number FC030576 and branch number BR015634 at the address 1st Floor, 24 Hills Road, Cambridge CB2 1JP | August 2014

- Avoid stating that data files are "available upon request"

Please indicate the figures or tables for which source data files have been provided:

For fish analyses:
- Figure 1: Excel "CPP_varenicline and bupropion"
- Figure 2: Excel "Figure_2_Heritability_CPP"
- Figure 3-4: Excel "Figure_3-4_CPP_sa1569-sa202"
- Figure 6: Excel "Figure_6_Habituation_amisulpride"
- Figure 7: Excel "Figure_7_CPP_amisulpride"
- Figure 8: Excel "Figure_8_qPCR"

For human analyses: Supplementary material and Supplementary Table 3.
